# Cardiovascular Risk in Patients with Chronic Obstructive Pulmonary Disease: A Systematic Review

**DOI:** 10.3390/jcm13175173

**Published:** 2024-08-31

**Authors:** Ana Sá-Sousa, Cidália Rodrigues, Cristina Jácome, João Cardoso, Inês Fortuna, Miguel Guimarães, Paula Pinto, Pedro Morais Sarmento, Rui Baptista

**Affiliations:** 1 MTG Research and Development Lab, 4200-604 Porto, Portugal; 2 Center for Health Technology and Services Research-CINTESIS@RISE, MEDCIDS-Department of Community Medicine, Information and Health Decision Sciences, Faculty of Medicine, University of Porto, 4200-450 Porto, Portugal; 3 Pulmonology Department, Unidade Local De Saúde de Coimbra, 3004-561 Coimbra, Portugal; 4 Pulmonology Department, Unidade Local de Saúde São José, 1150-199 Lisboa, Portugal; 5 NOVA Medical School, Nova University Lisbon, 1169-056 Lisboa, Portugal; 6 Pulmonology Department, Unidade Local de Saúde de Gaia e Espinho, 4434-502 Vila Nova de Gaia, Portugal; 7 Chest Department, Unidade Local De Saúde de Santa Maria, 1649-035 Lisboa, Portugal; paulagpinto@gmail.com; 8 Environmental Health Institute (ISAMB), Faculty of Medicine, University of Lisbon, 1649-028 Lisboa, Portugal; 9 Department of Internal Medicine, Heart Failure Day Hospital, Hospital da Luz de Lisboa, 1500-650 Lisboa, Portugal; 10 Department of Cardiology, Unidade Local De Saúde de Entre o Douro e Vouga, 4520-211 Santa Maria da Feira, Portugal; 11 Faculty of Medicine, University of Coimbra, 3000-075 Coimbra, Portugal; 12 Center for Innovative Biomedicine and Biotechnology (CIBB), University of Coimbra, 3000-548 Coimbra, Portugal; 13 Clinical Academic Center of Coimbra (CACC), 3004-531 Coimbra, Portugal

**Keywords:** chronic obstructive pulmonary disease, comorbidities, mortality, cardiovascular disease, myocardial infarction, cardiopulmonary risk

## Abstract

**Background/Objectives**: A comprehensive and up-to-date review on cardiovascular disease (CVD) risk in patients with COPD is needed. Therefore, we aimed to systematically review the risk of a range of CVD in patients with COPD. **Methods**: We searched three databases (Pubmed, Web of Science, SCOPUS) from inception to September 2023 using terms related to COPD and CVD. Observational studies were included if they (1) were conducted in adults with a diagnosis of COPD based on the GOLD criteria, spirometry, physician diagnosis, or review of electronic health records; (2) reported the risk of CVD, namely of myocardial infarction (MI), ischaemic heart disease (IHD), atrial fibrillation (AF), heart failure, cerebrovascular disease, pulmonary hypertension, and peripheral vascular disease, compared with a control population using a measure of risk. A narrative synthesis was used. **Results**: Twenty-four studies from 2015 to 2023, mainly from Europe (*n* = 17), were included. A total of 3,485,392 patients with COPD (43.5–76.0% male; 63.9–73.5 yrs) and 31,480,333 (40.0–55.4% male, 49.3–70.0 yrs) controls were included. A higher risk of CVD in patients with COPD was evident regarding overall CVD, MI, IHD, heart failure, and angina. Higher risks of arrhythmia and AF, stroke, sudden cardiac death/arrest, pulmonary embolism, pulmonary hypertension, and peripheral vascular disease were also found, although based on a small amount of evidence. **Conclusions**: Patients with COPD have a higher risk of CVD than the general population or matched controls. This review underscores the need for vigilant and close monitoring of cardiovascular risk in individuals with COPD to inform more precise preventive strategies and targeted interventions to enhance their overall management.

## 1. Introduction

Chronic obstructive pulmonary disease (COPD) is a heterogeneous lung disease characterized by chronic respiratory symptoms due to abnormalities of the airways and/or alveoli that cause persistent, often progressive, airflow obstruction [1]. Worldwide, 391.9 million people aged 30–79 years have COPD [2]. In 2019, COPD accounted for 3.3 million deaths and 74.4 million disability-adjusted life years (DALYs) [3]. COPD imposes a significant economic and human burden on individuals, healthcare systems, and societies.

The presence of comorbidities often complicates the management of COPD and acute exacerbations, influencing prognosis and requiring specific treatment [1]. The presence of comorbidities is linked to reduced health-related quality of life and increased morbidity, heightened susceptibility to hospital admissions, and an elevated risk of mortality [4].

Cardiovascular diseases (CVD) are one of the most important comorbidities of COPD [1,5], yet the overlap between these conditions often goes unrecognized. Research efforts have been made to unravel the key mechanisms behind the bidirectional link between COPD and CVD. Both conditions are related to the same risk factors (e.g., smoking, ageing, and physical inactivity) and to the pathophysiological mechanisms (e.g., arterial stiffness, inflammation, and endothelial dysfunction) [1]. Chronic low-grade systemic inflammation is a potential link between the two conditions. Triggers like air pollutants and cigarette smoking increase inflammatory processes. Moreover, the paradoxical effects of cardiac hormones (e.g., brain natriuretic peptide-BNP), released in response to altered myocardial wall stress, further contribute to the intricate relationship between CVD and COPD [6]. Lung hyperinflation, hypoxaemia, pulmonary hypertension, and shared genetics are other examples of the pathophysiological mechanisms underlying the association between COPD and CVD [7].

The presence of CVD is associated with poor prognosis, mainly an increased risk of exacerbations, hospitalizations, and mortality [1,5,8,9]. Routine cardiovascular assessments, including medical history, physical examination, cardiovascular risk scoring, diagnostic tests (e.g., echocardiogram, chest X-ray, BNP testing, etc.), have been indicated for the early identification of signs of concomitant CVD in patients with COPD [9]. Over the past decade, there have been several systematic reviews on the relation between COPD and CVD, with high heterogeneity [10,11,12,13,14,15]. The main differences were related to population (stable and exacerbated COPD) and CVD outcomes. The older studies covered an extensive range of CVD outcomes [10,11] and the most recent have focused on specific outcomes (myocardial infarction [12], coronary heart disease [13], stroke [14], and myocardial infarction, cardiovascular death, and stroke) [15]). In addition, some reviews set cut-offs for the sample size (50, 100) [11,15] and limited the outcomes to specific risk measures (e.g., hazard ratio) [13] as inclusion criteria. A comprehensive and up-to-date review of CVD risk in patients with COPD is needed.

Therefore, we aimed to systematically review the risk of a range of CVD in patients with COPD.

## 2. Materials and Methods

### 2.1. Study Design

This systematic review was reported according to the preferred reporting in systematic reviews and meta-analysis (PRISMA) guidelines [16]. We have registered the review with PROSPERO (registration number: CRD42023491294).

### 2.2. Search Strategy

We searched three databases (Pubmed, Web of Science, SCOPUS) from inception to September 2023. The search strategy included terms related to COPD and CVD. All studies were uploaded into EndNote to remove duplicates.

### 2.3. Eligibility Criteria

Observational studies were included if they met the following criteria: (1) conducted in adults with a diagnosis of COPD based on the GOLD criteria, spirometry, physician diagnosis, or review of electronic health records (diagnosis codes, medications prescribed, healthcare use); (2) reported the risk of CVD compared with a control population (non-COPD population, matched control, general population). Risk had to be reported as a measure of risk (e.g., hazard ratio, rate ratio, risk ratio, odds ratio, incidence rate ratio). The CVD outcomes considered were mortality, hospitalization or morbidity related to myocardial infarction (MI); ischaemic heart disease (IHD); atrial fibrillation (AF); heart failure; cerebrovascular disease; pulmonary hypertension; and peripheral vascular disease. These outcomes were considered as defined in the original studies, but had to be identified through physician diagnosis, clinical measurements, or review of electronic medical records (diagnosis codes, medications prescribed, health care use). We excluded studies that were based on a self-reported diagnosis of COPD or CVD, included an intervention, and used animals. Reviews, non-research letters, abstracts, case reports, conference proceedings, theses, and books were also excluded.

### 2.4. Screening, Selection Process, and Data Extraction

Studies were uploaded to Rayyan software (https://www.rayyan.ai/) for title and abstract independent screening by two reviewers (C.J. and I.F.). Any disagreement was resolved by discussion with a third author (A.S.-S.). Full-text articles were then read independently by two authors (C.J. and A.S.-S.) to identify studies that met the inclusion criteria. The two reviewers used a standardized form to independently extract data from each article, including the author’s surname and year of publication, country, study design and setting, diagnostic criteria, population, and CVD risk.

### 2.5. Data Synthesis

Narrative synthesis was used to report the results. This was considered the most appropriate approach given the heterogeneity of data between the included studies. The findings were initially drafted by one researcher (C.J.) and then reviewed by a second researcher (A.S.-S.).

## 3. Results

### 3.1. Study Selection

**Figure 1:**
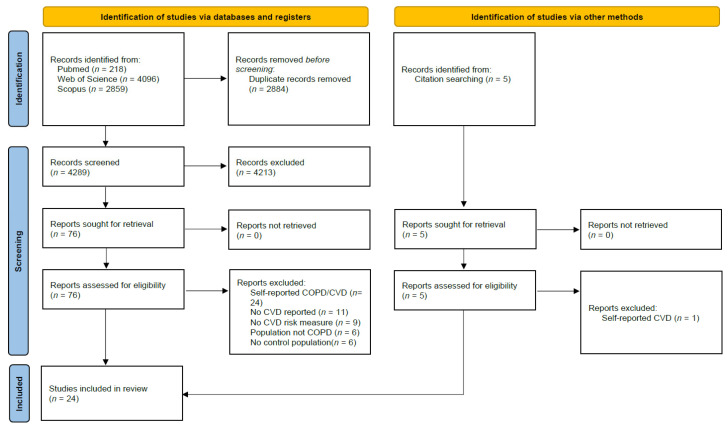
PRISMA flowchart.

The database search yielded 7173 studies. After removing duplicate results, 4289 articles were screened for relevant content. During title and abstract screening, 4213 articles were excluded. Finally, 76 articles were full-text screened and 56 were excluded. Four additional articles were included through screening of previous reviews. In total, 24 studies were selected for qualitative analysis (Figure 1).

### 3.2. Characteristics of Included Studies

A summary of the included studies is shown in Table 1. The studies are from 2015 to 2023, mainly from Europe (*n* = 17), with the UK being the most represented country (*n* = 5). The remaining studies were conducted in North America (*n* = 5 Canada, *n* = 1 USA) and Asia (*n* = 1 Korea). A total of 19 studies were cohorts (15 retrospective and of those 8 case-control) and 5 were cross-sectional (1 case-control). The sample size of the studies ranged from 775 to 7,419,791. A total of 3,485,392 patients with COPD (43.5 to 76% male) and 31,480,333 (40 to 55.4% male) controls were included in the studies. Two studies did not report the number of controls included [17,18]. The mean age of the participants ranged from 63.9 to 73.5 years in the COPD population and from 49.3 to 70.0 in the control population. Two studies did not report the mean age of participants (buts instead median age or relative frequencies of age groups [18,19,20,21,22,23,24]) and two did not report age [17,25].

All the included studies used data from either routine clinical, administrative, or research databases. COPD was defined in 18 studies using diagnostic codes (ICD-8/-9-10 in 13 studies), either alone or in combination with other criteria [23,36,38,39]. Other criteria required that patients with COPD were of a certain age (≥35 y, ≥40 y, ≥55 y, or 40–79 y [17,19,20,21,26,29,32,35,37,38]), had been prescribed COPD medication [17,19,26,34,39,40], had spirometry data [22,24,33,37,39,40], had >10 pack-years [37], and had participated in a COPD management program [40]. Most studies defined cardiovascular diseases using diagnostic codes (*n* = 22), with only two studies relying on specialist confirmation [22,33].

A summary of the risks of the different CVDs, either related to mortality or presence of comorbidity are presented in Table 2 and Table 3, respectively, and described below.

### 3.3. CVD

Six studies analyzed the risk of CVD mortality, and five found a higher risk in patients with COPD compared to the general population (HR 1.53 [24]; RR 1.84–2.43 [17,19,23,26]). The same pattern was observed when analyzing the risk (RR 1.89–2.17 [17,19,26,32]) and the odds (OR 1.83–2.09 [19,39]) of having CVD.

### 3.4. MI

Four out of five studies found a higher risk of MI mortality in patients with COPD compared to patients without COPD (HR 1.25 [35]; RR 1.51–1.63 [17,19,25,26]). Eight studies analyzed the risk of MI and six found similar risks (HR 1.26 and 1.47 [28,31]; RR 1.18–2.25 [17,23,26,35]). The same was true when an odds ratio was used (OR 1.40–2.91) [19,21,27,29]. Two other studies analyzed the risk of MI by age group and found a higher risk in younger patients with COPD (HR 10.34 and 3.15) [18,20].

### 3.5. IHD

COPD was associated with a higher risk of death from IHD in three studies (HR 1.03 [36], RR 1.91–2.68 [17,23]). COPD was also associated with a higher risk of having this comorbidity (HR 1.34–1.80 [37], RR 1.41–2.36 [17,30,35,38,40]). This association was also seen when using odds ratios (OR 1.74–2.32) [29,36,39].

### 3.6. Heart Failure

Evidence from five studies showed that COPD was associated with an increased risk of death from heart failure (HR 1.65 [36], RR 2.48–4.09 [17,19,25,26]). Eleven studies showed that the risk of heart failure was higher in patients with COPD, with most studies showing a two-fold higher risk compared to controls (HR 1.51–6.80 [18,37], RR 2.41–3.75 [17,19,23,25,26,30,35,38,40]). This association was also found in four studies using odds ratios (OR 2.17–5.21) [19].

### 3.7. Angina

Patients with COPD had a higher risk of having angina when compared with controls. This was found in five studies (HR 1.27–3.8, RR 1.98–2.08) [18,19,23,26,35]. Curkendall et al. also found this association (OR 1.86) [19].

### 3.8. Arrhythmia and AF

One study analyzed the risk of death from arrhythmia (RR 2.81, 95%CI 1.59–4.98 [19]) and a second from AF (HR 1.08, 95%CI 1.06–1.10 [36]) in patients with COPD, both with significant results. When analyzing the risk (RR 1.56–1.98 [23,26,40]) and odds (OR 1.39–1.70 [29,36]) of having AF, the same pattern was observed. Morgan et al. showed that the risk of AF was higher in younger patients with COPD when compared with older age groups (HR 3.94 vs 1.31) [18].

### 3.9. Stroke

Six studies analyzed the risk of dying from a stroke, and four showed a higher risk (HR 1.84, 95%CI 1.80–1.89 [36]; RR 1.24–1.84 [17,23,26]) in patients with COPD. Five studies showed a higher risk of stroke in patients with COPD (HR 1.15–3.81 [18], RR 1.33–1.71 [17,23,26,35]). The studies that reported odds ratios were not significant, except for Curkendall et al. (OR 1.24) [19].

### 3.10. Sudden Cardiac Death/Arrest

Patients with COPD had a higher risk of sudden cardiac death (HR 1.93 [33]) and cardiac arrest (HR 1.87–4.06 [18]) compared with controls

### 3.11. Pulmonary Embolism

Two studies analyzed the risk of dying from a pulmonary embolism, but the results were non-significant [19,26]. Three studies reported results on the risk of pulmonary embolism, but only two showed a higher risk associated with COPD (RR 2.39 and 2.72) [26,35]. The same was observed when using odds ratios (OR 2.51 and 5.47) [19,27].

### 3.12. Pulmonary Hypertension

Two studies analyzed the presence of pulmonary hypertension in patients with COPD. Morgan et al. showed that the risk of this condition was higher in patients with COPD, decreasing with age (HR 3.70–27.70) [18]. Baty et al. 2013 also found higher odds in patients with COPD (OR 5.60–5.80) [29].

### 3.13. Peripheral Vascular Disease

Both the risk of death from peripheral vascular disease (HR 1.32 [36]) and the risk of having PVD (HR 1.42–7.70 [18]) were higher in patients with COPD. In patients with COPD, the odds of having PVD was found to be 1.85–2.80 [29,36].

**Figure 2:**
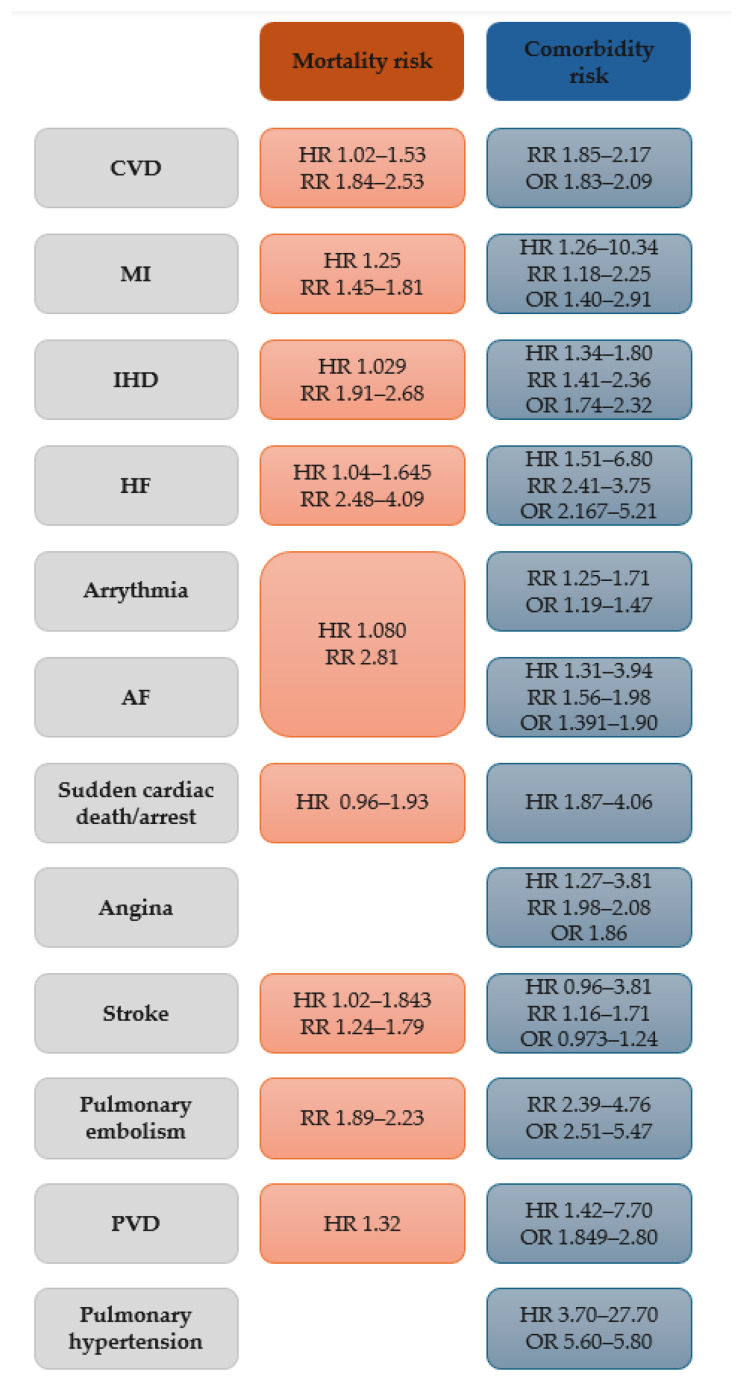
Measures of mortality and comorbidity risk in patients with COPD. Abbreviations: AF, atrial fibrillation; CVD, cardiovascular disease; HF, heart failure; HR, hazard ratio; IHD, ischemic heart disease; MI, myocardial infarction; OR, odds ratio; PVD, peripheral vascular disease; RR, rate ratio/relative risk.

Figure 2 presents a summary of risks related to mortality and comorbidity.

## 4. Discussion

Most studies that have examined the risk of CVD in patients with COPD suggest that those with COPD have a higher risk than the general population or matched controls. This review has also highlighted the heterogeneity in the criteria used to diagnose COPD among the published studies.

### 4.1. CVD

Eight studies analyzed the risk of CVD (either as cause of death or as a comorbidity) [17,19,23,24,26,32,39] and seven found a higher risk in patients with COPD compared with the general population/matched controls. The only study that did not show a significant association was the study conducted in an Asian population and this difference may be due to the characteristics of the population studied and the specific epidemiological features [41]. Further research needs to be conducted to better understand the link between COPD and CVD [42], mainly in regions that are less represented in the collected evidence, such as Asia, South America, and Africa.

### 4.2. MI, IHD, and Angina

COPD has been associated with a higher risk of MI [17,19,23,25,26,28,31,35], IHD [17,23,29,30,35,36,38,39,40], and angina [18,19,23,26,35]. The only study that did not find a significant association between MI (death or event) was Rodriguez et al., which may be in part explained by the exclusion of patients with a history of coronary heart disease [25]. This evidence is in line with a previous review [12]. These consistent findings across multiple studies suggest a robust association between COPD and an increased susceptibility to CVD, underscoring the need for vigilant monitoring of cardiovascular health as well as cardiopulmonary risk in individuals with COPD [9,43]. Assessment of blood eosinophil count together with commonly used forced expiratory volume in 1 s (FEV1) can be of value for monitoring patients with COPD. Different incidences of non-fatal MI and cardiovascular death across distinct pharmacological treatments were found, which were more pronounced with increasing blood eosinophil [44]. Future research could delve deeper into the underlying mechanisms of this association to inform more precise preventive strategies and targeted interventions to enhance the overall management of COPD patients [42].

### 4.3. Heart Failure

Evidence is robust showing that COPD is associated with an increased risk of heart failure [17,18,19,23,25,26,30,35,36,37,38,40], with most studies showing more than a two-fold higher risk compared to controls. This has been a topic of interest in recent opinion reviews [43,45,46]. The EURObservational Research Programme Heart Failure Long-Term Registry found that up to 19% of people hospitalized with heart failure also had diagnosed COPD [47]. To reduce symptoms, delay progression, and improve prognosis, it is essential to better screen and diagnose these two coexisting conditions and when present, to establish a management strategy that addresses both simultaneously. This is especially true given the rising mortality rates and the significant negative impact of each disease on quality of life and functional status [48].

### 4.4. Other CVD Outcomes

Seven studies have shown the link between stroke and COPD [17,18,19,23,26,35,36], with consistent risk measures. These results are aligned with a recent systematic review on the topic, which showed pooled odds for stroke risk of 1.40 (95% CI 1.24–1.59) and for stroke mortality of 1.20 (95% CI 1.13–1.27) [14]. Evidence from other CVD outcomes is still scarce, with too few studies to allow a proper synthesis of the associated risk. Future observational cohort studies should gather more information regarding these outcomes.

### 4.5. Strengths and Limitations

This review benefits from the use of a comprehensive search strategy that included three bibliographic databases. Our review used more robust diagnostic criteria for both COPD and CVD, thus excluding several studies previously presented in prior reviews. Nevertheless, the risk for each CVD was summarized as presented in the original studies, but significant heterogeneity in CVD definitions may exist across these studies, which were not accounted for. Patients with COPD were mostly selected based on diagnosis codes using internationally standardized classifications, but considering that COPD is frequently underdiagnosed, more symptomatic/severe COPD are likely to be over-represented in the original studies included. In addition, some studies may have excluded a proportion of patients with COPD from their analyses by using stricter criteria such as a specific age range (excluding either younger or older patients) or by requiring spirometry data, which is not always available. Validation and comparison of the accuracy of CVD and COPD case definitions [49,50] in distinct databases can be a strategy to overcome these limitations in the future [51]. The severity of COPD and COPD phenotypes may also play a role in the development of CV comorbidities as has been previously suggested [52]. However, this could not be determined. Only the study from Ingebrigtsen presented the risk in relation to GOLD grades. With the increasing number of COPD diagnoses based on spirometry [53], future studies may shed light on this unanswered research question [42]. Another limitation is that most studies did not adjust the risk of CV outcomes for patients’ smoking status. This is probably because this information was not available in several databases. However, six studies included this adjustment, three of which used primary care databases and three published since 2020. This shows that the availability and quality of reporting of this information is likely to be improving, which will allow future observational studies to better adjust for possible confounders and clarify the role of COPD in the development of CVD. It also highlights the potential of using large datasets that include EMR from primary and secondary care [54]. In addition, we did not account for potential duplication of findings, as many studies were the sole representatives from a country or derived from the same database but assessed distinct outcomes. However, it is important to note that in studies conducted in the UK and Canada, despite the majority utilizing different databases and/or index periods and focusing on distinct mortality and comorbidity risks, some overlap in findings may still exist. This will need to be better considered in future systematic reviews including meta-analysis with sensitivity analysis. As this was primarily a qualitative synthesis, with the aim of summarizing all existing evidence on the topic, the risk of bias of the included studies was not assessed.

### 4.6. Clinical Implications and Research Future Directions

To address the coexistence of COPD and CVD a comprehensive and integrated care model is advocated, encompassing primary prevention, screening in primary care settings, and fostering of multidisciplinary collaboration (pulmonologists, cardiologists, and general practitioners) in both primary and secondary care [6,42]. CVD early detection can be performed through minimal checkup (e.g., medical history, physical examination, blood tests, cardiovascular risk scoring) and, if further investigation is needed, through predictive biomarkers (e.g., BNP) and imaging procedures, such as echocardiography and coronary computed tomography angiography [9,55]. Clinical decision support systems are also recommended as a pivotal strategy [6]. These collaborative efforts are crucial for stratifying cardiovascular risk in COPD patients ensuring the delivery of appropriate treatment. The higher CVD risk emphasizes also the burden of cardiopulmonary risk in patients living with COPD [43,56]. Recognizing that exacerbations pose a significant threat to both pulmonary and CV health, there is an imperative need for proactive identification and targeted treatment of COPD individuals at risk of exacerbation. It would be of benefit if, in the near future, we could quantify and categorize levels of cardiopulmonary risk in patients with COPD. Well-designed retrospective and longitudinal real-world studies including electronic health records from primary and secondary care are needed to comprehensively address this research question [42]. The potential of large datasets will probably enhance the quality and comprehensiveness of the data collected. As diagnosis codes are associated with some misclassification, future studies may consider the presence of COPD only when related outcomes are assessed (e.g., FEV1). As data availability on smoking habits and COPD severity improves, future studies should provide clearer insights into their correlation with CVD. This review focused on the risk of CVD in COPD in a unidirectional way. Yet, as COPD also adversely affects the prognosis of CVD, future reviews could include both angles of this bidirectional relationship.

## 5. Conclusions

Most studies which have investigated the risk of CVD in patients with COPD suggest that those with COPD have a higher risk than the general population or matched controls. This review underscores the need for vigilant and close monitoring of cardiovascular and cardiopulmonary risk in individuals with COPD to inform more precise preventive strategies and targeted interventions to enhance their overall management. The retrospective design of most studies, coupled with limited or no adjustments for confounding factors, impairs the drawing of definitive conclusions regarding CV risk in patients with COPD. Additional well-designed prospective studies to comprehensively address this question are needed.

## Figures and Tables

**Table 1:** Characteristics and results of included studies.

Author, Year	Country	Design	Source, Period	Diagnostic Criteria	COPD	Control	Cardiovascular Risk
Huiart et al., 2005 [17]	Canada	Retrospective Cohort	Health insurance Saskatchewan databases, 1990–1997	COPD ≥55 y ≥3 prescriptions of COPD medications CVD Death and Hospitalizations (ICD-9 codes)	5648 53.9% male 73.5 (9.6) y	NS (general population)	**Mortality–RR (95%CI) adjusted for age, gender, and year** CVD 1.95 (1.83–2.07) MI 1.63 (1.41–1.86) IHD 1.91 (1.74–2.09) HF 2.83 (2.32–3.33) Cerebrovascular disease 1.60 (1.36–1.85) **Hospitalization–RR (95%CI) adjusted for age, gender, and year** CVD 1.89 (1.83–1.94) MI 1.30 (1.15–1.44) IHD 1.47 (1.38–1.56) HF 3.07 (2.91–3.23) Cerebrovascular disease 1.27 (1.16–1.38)
Sidney et al., 2005 [26]	USA	Retrospective case-control cohort study	Kaiser Permanente Medical Care Program of Northern California, 1996–1999	COPD ≥40 y Hospitalization/outpatient visit with diagnosis of COPD-ICD-9 codes ≥2 prescriptions for COPD medications CVD Death and Hospitalizations (ICD-9/-10 codes)	45,966 55.4% male 64.4 (12.2) y	45,966 55.4% male 64.4 (12.2) y	**Mortality–RR (95%CI) adjusted for age, gender, HTA, hyperlipidemia, and diabetes** All CVD 1.84 (1.70–1.98) MI 1.81 (1.54–2.12) HF 3.53 (2.38–5.25) Stroke 1.25 (1.03–1.51) Pulmonary embolism 1.89 (0.93–3.85) Other CVD 1.96 (1.77–2.16) **Hospitalizations–RR (95%CI) adjusted for age, gender, HTA, hyperlipidemia, and diabetes** Any CVD 1.95 (1.88–2.03) MI 1.89 (1.71–2.09) VT/VF/cardiac arrest 2.80 (1.87, 4.20) Atrial fibrillation 1.98 (1.73–2.25) Other arrhythmia 1.71 (1.43–2.03) Angina 1.98 (1.73–2.27) HF 3.75 (3.39–4.15) Stroke 1.33 (1.21–1.47) Pulmonary embolism 2.72 (2.00–3.68) Other CVD 1.85 (1.73–1.97)
Curkendall et al., 2006 [19]	Canada	Retrospective cohort	Saskatchewan Health Database, 1998–2001	COPD ≥40 y ≥2 prescriptions of COPD medications Physician claims/hospital data with diagnosis of COPD-ICD-9 codes CVD Inpatient/outpatient diagnoses (ICD-9/-10 codes)	11,493 54% male 83% ≥ 65 y	22,986 54% male 80% ≥ 65 y	**Mortality–RR (95%CI) adjusted for age and sex** Any CV 2.07 (1.82–2.36) MI 1.51 (1.14–2.01) Arrhythmia 2.81 (1.59–4.98) HF 4.09 (2.64–6.33) Stroke 1.24 (0.90–1.71) Pulmonary embolism 2.23 (0.60–8.23) **Hospitalizations–RR (95%CI) adjusted for age, sex, history of CV events, diabetes, HTA, and hypercholesterolemia** Any CV 2.17 (2.00–2.33) MI 1.49 (0.71–3.13) Arrhythmia 1.67 (1.27–2.22) Angina 2.08 (1.52–2.86) HF 3.45 (2.78–4.17) Stroke 1.23 (0.68–2.22) Pulmonary embolism 4.76 (0.79–25.00) **Morbidity–OR (95%CI) adjusted for age, sex, history of CV events, diabetes, HTA, and hypercholesterolemia** MI 1.83 (1.64–2.05) Arrhythmia 2.09 (1.96–2.23) Angina 1.86 (1.72–2.01) HF 5.21 (4.86–5.58) Stroke 1.24 (1.15–1.34) Pulmonary embolism 5.47 (4.25–7.03) Other CVD 2.09 (1.99–2.20)
Feary et al., 2010 [20]	UK	Cross-sectional	Health Improvement Network, 2005–2007	COPD ≥35 y Primary care EMR -disease codes CVD Primary care EMR -disease codes	29,870 48.1% male 70.7% ≥ 65 y	1,174,240 51.4% male 27.8% ≥ 65 y	**HR (95%CI) adjusted for sex and smoking status** MI: ≥75 y 1.34 (1.03–1.73) to 35–44 y 10.34 (3.28–32.6) Stroke: ≥75 y 1.10 (0.98–1.23) to 35–44 y 3.44 (0.85–13.84)
Schneider et al., 2010 [27]	UK	Retrospective cohort and nested case– control	General Practice Research Database, 1995–2005	COPD 40–79 y Primary care EMR -disease codes CVD Primary care EMR -disease codes	35,772 51.3% male 73.4% ≥ 60 y	35,772 51.3% male 73.4% ≥ 60 y	**OR (95%CI)** **Adjusted for smoking status, BMI, HTA, hyperlipidemia, diabetes, and NSAID use** MI 1.40 (1.13–1.73) **Adjusted for smoking status, HTA, beta agonist use, xanthine use, quinolone use, macrolide use, vitamin K antagonist use and use of beta blockers, calcium channel blockers, diuretics, cardiac glycosides, and coronary dilatators** Arrhythmia 1.19 (0.98–1.43) **Adjusted for smoking status, BMI, HTA, aspirin use, and diabetes** Stroke 1.13 (0.92–1.38) **Adjusted for smoking status, BMI, HTA, and non-steroidal anti-inflammatory drugs use** Pulmonary embolism 2.51 (1.62–3.87) Deep vein thrombosis 1.35 (0.97–1.89)
Rodriguez et al., 2010 [25]	UK	Retrospective cohort and case-control	General Practice Research Database, 1996–2001	COPD Oxford Medical Information System (OXMIS) and Read codes CVD Diagnosis code	1927	16,546	**MI-RR (95%CI) adjusted for age and sex** Mortality (1st year) 1.45 (0.79–2.66) Event 1.18 (0.81–1.71) **HF-RR (95%CI) adjusted for age and sex** Mortality (30-days diagnosis) 2.48 (1.36–4.54) Hospitalization 2.81 (1.94–4.07) Diagnosis 2.94 (2.46–3.51)
Sode et al., 2011 [28]	Denmark	Retrospective cohort	Danish Patient Registry and Danish Causes of Death Registry, 1980–2006	COPD ICD-8/-10 codes CVD ICD-8/-10 codes	313,958 55% male M 55 (IQR 46–66) y	7,105,833 50% male M 26 (IQR 1.2–42) y	**HR (95%CI) adjusted for age, sex, descent, geographical residency, and level of education** MI 1.26 (1.25–1.27)
Baty et al., 2013 [29]	Switzerland	Retrospective case-control	Inpatient database of the Swiss Federal Office for Statistics, 2002–2010	COPD ≥ 40 y ICD-10 CVD ICD-10	1,609,317 65% male M 73 (IQR 64–80) y	2,989,359	**OR (95%CI) adjusted for age and sex** MI 1.80 (1.7–1.8) Other forms of IHD 2.10 (2.2.2) IHD, unspecified 2.10 (2–2.2) Atherosclerotic heart disease 1.70 (1.6–1.7) AF/flutter 1.90 (1.9–2) Persistent AF 1.70 (1.7–1.8) HF 3.20 (3.1–3.3) Left ventricular failure 2.50 (2.5–2.6) HF, unspecified 2.70 (2.6–2.8) Primary pulmonary hypertension 5.80 (5.5–6.1) Other secondary pulmonary hypertension 5.60 (5–6.3) Other pulmonary heart diseases 8.90 (8.1–9.8) Pulmonary heart disease, unspecified 16 (14–17) Peripheral vascular disease 2.80 (2.7–2.9) Atherosclerosis of arteries of extremities 2.20 (2.2–2.3) Venous insufficiency 2.10 (2–2.2)
Cazzola et al., 2012 [21]	Italy	Cross-sectional	Health Search Database, 2009	COPD >34 y ICD-9 codes CVD ICD-9 codes	25,281 72.5% ≥ 65 y	625,467 32.1% ≥ 65 y	**OR (95%CI)** **Adjusted for age and sex** MI 2.91 (95% CI 2.74–3.09) Angina and coronary disease, arrhythmia, HF, Cerebrovascular, pulmonary embolism, other heart disease–significant OR > 2
Garcia-Olmos et al., 2013 [30]	Spain	Cross-sectional	Family practice databases of Madrid Autonomous Region, 2007	COPD Expanded Diagnosis Clusters (EDCs) codes CVD EDCs codes	3183 76% male 71.41 (11.5) y	198,670 47.65% male	**RR(95%CI) adjusted for age and sex** IHD 1.41 (1.11–1.71) HF 2.41 (1.88–2.93) Arrhythmia 1.25 (1.05–1.44) Cardiac valve disease 1.25 (0.73–1.77) Cerebrovascular disease 1.16 (0.89–1.42)
Yin et al., 2014 [31]	Sweden	Retrospective cohort	Prescribed Drug, Patient, Cause of Death, Income, Educational, and Emigration Registers	COPD ICD-8/9/10 codes CVD ICD-8/9/10 codes	51,348 44.33% male 71.05 y	6,743,342 48.72% male 49.25 y	**HR (95%CI) adjusted for age, gender, education, income, and drugs prescription** MI 1.47 (1.41–1.55)
Gershon et al., 2015 [32]	Canada	Prospective cohort	Five Ontario health administrative databases, 1991–2008	COPD ≥35 y Physician diagnosis ≥1 hospitalization/ambulatory visit (ICD-9/10 code) CVD ICD-9/10 code	909,948 49.6% male 64.3 (13.8) y	6,331,643 47.8% male 53.4 (13.3) y	**RR adjusted for age, sex, income, rurality, asthma, and other comorbidities** CVD Hospitalizations 1.94
Lahousse et al., 2015 [33]	Netherlands	Prospective cohort	Rotterdam study database, 1990–2011	COPD FEV1/FVC < 70% (or by a physician based on the combination of clinical history, physical examination, and spirometry) CVD Based on EMR Confirmed by a cardiologist	1615 56% male 70 (13) y	11,856 40% male 63 (15) y	**HR (95%CI) adjusted for age, sex, and pack-years** Sudden cardiac death 1.93 (1.44–2.59)
Song et al., 2017 [34]	Korea	Retrospective cohort and case-control	National Health Insurance Service—National Sample Cohort, 2002–2013	COPD Prescription ≥ 1 of COPD medication ICD-10 codes CVD ICD-10 codes	11,755 47.4% male 64.1 (12.8) y	11,755 47.5% male 63.9 (13.3) y	**Mortality–HR(95%CI) adjusted for age, sex, HTA, diabetes, dyslipidemia, chronic kidney disease, end stage renal disease, previous MI, HF, current smoker, and stroke/TIA** Sudden cardiac 0.96 (0.68–1.38) CVD 1.02 (0.80–1.30)
Ställberg et al., 2018 [35]	Sweden	Retrospective cohort and case-control	Primary care EMR, 2000–2014	COPD ≥ 40 y ICD-10 CVD ICD-10	17,479 43.5% male 65 (11.90) y	84,514 43.5% male 65 (11.90) y	**Mortality–HR(95%CI)** IHD 1.25 (1.16–1.35) HF 1.04 (0.97–1.13) Stroke 1.02 (0.89–1.18) **Comorbidity–RR(95%CI)** MI 2.25 (2.15–2.35) IHD 2.36 (2.28–2.45) Angina 2.01 (1.94–2.09) HF 3.27 (3.17–3.37) Stroke—Cerebral infarction 1.71 (1.62–1.80) Stroke—Cerebral infarction 1.69 (1.53–1.87) Pulmonary embolism 2.39 (2.19–2.61)
Morgan et al., 2018 [18]	UK	Retrospective cohort and case-control	Primary care EMR (Clinical Practice Research Datalink-CPRD), 2004–2015	COPD CPRD validated algorithm (COPD specific read code, >1 prescription of COPD medication and spirometry) CVD Read codes	209,909 53% male 52.4% ≥ 65 y	NS (CPRD)	**Comorbidity–HR (95%CI) adjusted for sex and GP practice** MI: >85 y 1.30 (1.12–1.52) to 35–54 y 3.15 (2.64–3.76) Sudden cardiac arrest: >85 y 1.87 (1.20–2.93) to 35–54 y 4.06 (2.51–6.55) Angina: >85 y 1.27 (1.06–1.53) to 35–54 y 3.81 (3.35–4.34) AF: >85 y 1.31 (1.17–1.47) to 35–54 y 3.94 (3.22–4.81) HF: >85 y 1.51 (1.32–1.72) to 35–54 y 6.80 (5.60–8.25) Peripheral artery disease: >85 y 1.42 (1.16–1.73) to 35–54 y 7.70 (6.33–9.37) Stroke: >85 y 0.96 (0.82–1.12) to 35–54 y 3.81 (3.14–4.62) Pulmonary hypertension: >85 y 3.70 (2.20–6.23) to 35–54 y 27.70 (15.33–50.06)
Carter et al., 2019 [36]	UK	Retrospective cohort and case-control	National Health Service Local Health Authority computerized hospital activity analysis register, 2000–2013 National Health Tracing Services, 2013	COPD ICD-10 codes OPCS-4 codes CVD ICD-10 codes OPCS-4 codes	31,646 51% male 70.3 (12) y	158,230 51% male 70 (12) y	**Mortality–HR (95%CI) adjusted for age, sex, ethnic group, other CVD, cardiac procedures, and common causes of death** IHD 1.029 (1.009–1.050) AF 1.080 (1.057–1.103) HF 1.645 (1.608–1.684) Cerebrovascular 1.843 (1.796–1.891) Peripheral vascular disease 1.320 (1.271–1.371) **Morbidity–OR (95%) adjusted for age, sex, ethnic group, other CVD, cardiac procedures, and common causes of death** IHD 1.742 (1.688–1.797) AF 1.391 (1.339–1.444) HF 2.167 (2.081–2.255) Cerebrovascular 0.973 (0.921–1.027) Peripheral vascular disease 1.849 (1.737–1.969)
Ingebrigtsen et al., 2020 [37]	Denmark	Prospective cohort	Copenhagen General Population Study, 2003–2014 Danish Patient Registry, 1977–2014	COPD ≥40 y FEV_1_/FVC < 0.70 >10 pack-years CVD ICD-8/10 codes	7968 56% male GOLD 1–2 7447 56% male 66 (11) y GOLD 3–4 521 59% male 70 (9) y	30,555 40% male 59 (11) y	**Hospitalizations–HR (95%CI) adjusted for age, sex, smoking, CVD family history, physical activity, BMI, HTA, diabetes, and total and HDL cholesterol** **IHD** GOLD 1–2 1.34 (1.20–1.49) GOLD 3–4 1.80 (1.42–2.27) **HF** GOLD 1–2 1.91 (1.63–2.26) GOLD 3–4 2.94 (2.22–3.88)
Cave et al., 2021 [38]	Canada	Retrospective cohort	Canadian Primary Care Sentinel Surveillance Network (CPCSSN), 2013–2017	COPD ≥40 y CPCSSN definition (based on ICD-9 and ATC medication codes) CVD ICD-9 codes	4629 48% male 62.8 (11.2) y	128,342 42.1% male 55.8 (10.9) y	**RR (95%CI) adjusted for age, sex, smoking status, and urban/rural postal code** IHD 1.44 (1.35–1.55) HF 2.64 (2.40–2.90)
Jurevičienė et al., 2022 [39]	Lithuania	Cross-sectional	National Health Insurance Fund database (primary and secondary care), 2012–2014	COPD 40–79 y Spirometry COPD medication ICD-10 codes CVD ICD-10 codes	4834 69.1% male 67.2 (8.4)	316,463 40.9% male 63.6 (10.1)	**OR (95%CI) adjusted for sex, age, and place of residence** CVD 1.83 (1.69–1.97) IHD 2.32 (2.14–2.50) Arrhythmia 1.47 (1.38–1.55) HF 2.61 (2.46–2.78)
Groenewegen et al., 2022 [40]	Netherlands	Retrospective cohort	Primary care data-Julius General Practitioners’ Network, 2014–2019	COPD Prescription for COPD medication Spirometry Participation in a COPD management program ICPC-codes CVD ICPC-codes	6223 50.3% male 64.1 (9.3) y	137,028 49.6% male 55.3 (10.6) y	**RR (95%CI) adjusted for age and sex** IHD 1.69 (1.49–1.92) AF 1.56 (1.38–1.77) HF 2.96 (2.58–3.40)
Svendsen et al., 2022 [22]	Norway	Cross-sectional, case-control	MicroCOPD, 2012–2015 GeneCOPD, 2003–2004	COPD Spirometry Physician confirmation CVD Coronary stenosis assessed by coronary computed tomography angiography Lumen reduction > 50% Specialist confirmation	347 54.2% male 43.5 ≥ 70 y	428 54.7% male 23.4 ≥ 70 y	**OR (95%CI) adjusted for age, body composition, pack-years, C-reactive protein, statin use, ACE inhibitors or ARB use, and diabetes** Coronary stenosis 1.80 (0.86–3.78)
Mattila et al., 2023 [24]	Finland	Prospective cohort	Health examination survey, 2000–2001	COPD FEV1/FVC <0.7 CVD ICD-10 codes	151 71.5% male 63.9 (range 35–89) y 49% ≥ 65 y	5922 45.8% male 21.5 ≥ 65 y	**Mortality–HR (95%CI) adjusted for ex, age, smoking, education level, BMI, leisure time physical activity, CVD, diabetes, and HTA** CVD 1.53 (1.08–2.16)
Maclagan et al., 2023 [23]	Canada	Retrospective cohort	Health administrative data, EMR, Immigration, Refugees and Citizenship Canada Permanent Resident Database, Canadian Community Health Survey, 2008–2016	COPD ≥40 y 1 COPD hospitalization and/or 3 physician claims ICD-10 codes Ontario Health Insurance Plan codes CVD ICD-9/10 codes Ontario Health Insurance Plan codes	152,125 48.1% male 72.9% ≥ 60 y	5,626,596 47.2% male 33% ≥ 60 y	**Mortality–RR (95%CI) adjusted for age and sex** CVD 2.43 (2.35–2.49) IHT 2.68 (2.59–2.77) Stroke 1.79 (1.67–1.91) **Comorbidities–RR (95%CI) adjusted for age and sex** MACE 1.98 (1.96–2.01) MI 1.88 (1.81–1.96) HF 2.82 (2.70–2.91) AF 1.79 (1.75–1.83) Unstable angina 2.05 (1.94–2.18) Stroke 1.71 (1.65–1.78)

Abbreviations: AF, atrial fibrillation; BMI, body mass index; EMR, electronic medical records; GP, general practice; HF, heart failure; HTA, arterial hypertension; ICD, International Classification of Disease; ICPC, International Classification of Primary Care; IHD, ischemic heart disease; IQR, interquartile range; M, median; MACE, major adverse cardiovascular events; MI, myocardial infarction; NS, not specified; OPCS-4, Office of Population Censuses and Surveys Classification of Interventions and Procedures; RR, rate ratio/relative risk; TIA, transient ischemic accident; VF, ventricular fibrillation; VT, ventricular tachycardia.

**Table 2:** Measures of mortality risk in patients with COPD.

Author, Year	CVD	MI	IHD	HF	Arrhythmia/AF	Sudden Cardiac Death	Stroke	Pulmonary Embolism	PVD
Huiart et al., 2005 [17]	RR 1.95 (1.83–2.07)	RR 1.63 (1.41–1.86)	RR 1.91 (1.74–2.09)	RR 2.83 (2.32–3.33)			RR 1.60 (1.36–1.85)		
Sidney et al., 2005 [26]	All RR 1.84 (1.70–1.98) Other RR 1.96 (1.77–2.16)	RR 1.81 (1.54–2.12)		RR 3.53 (2.38–5.25)			RR 1.25 (1.03–1.51)	RR 1.89 (0.93–3.85)	
Curkendall et al., 2006 [19]	Any RR 2.07 (1.82–2.36)	RR 1.51 (1.14–2.01)		RR 4.09 (2.64–6.33)	RR 2.81 (1.59–4.98)		RR 1.24 (0.90–1.71)	RR 2.23 (0.60–8.23)	
Rodriguez et al., 2010 [25]		RR 1.45 (0.79–2.66)		RR 2.48 (1.36–4.54)					
Lahousse et al., 2015 [33]						HR 1.93 (1.44–2.59)			
Song et al., 2017 [34]	HR 1.02 (0.80–1.30)					HR 0.96 (0.68–1.38)			
Ställberg et al., 2018 [35]		HR 1.25 (1.16–1.35)		HR 1.04 (0.97–1.13)			HR 1.02 (0.89–1.18)		
Carter et al., 2019 [36]			HR 1.029(1.009–1.050)	HR 1.645 (1.608–1.684)	HR 1.080 (1.057–1.103)		HR 1.843 (1.796–1.891)		HR 1.320 (1.271–1.371)
Mattila et al., 2023 [24]	HR 1.53 (1.08–2.16)								
Maclagan et al., 2023 [23]	RR 2.43 (2.35–2.49)		RR 2.68(2.59–2.77)				RR 1.79 (1.67–1.91)		

Abbreviations: AF, atrial fibrillation; CVD, cardiovascular disease; HF, heart failure; HR, hazard ratio; IHD, ischemic heart disease; MI, myocardial infarction; PVD, peripheral vascular disease; RR, rate ratio/relative risk.

**Table 3:** Measures of comorbidities risk in patients with COPD.

Author, Year	CVD	MI	IHD	HF	Arrhythmia	AF	Sudden Cardiac Arrest	Angina	Stroke	Pulmonary Embolism	Pulmonary Hypertension	Peripheral Vascular Disease
Huiart et al., 2005 [17]	RR 1.89 (1.83–1.94)	RR 1.30 (1.15–1.44)	RR 1.47 (0.38–1.56)	RR 3.07 (2.91–3.23)					RR 1.27 (1.16–1.38)			
Sidney et al., 2005 [26]	Any RR 1.95 (1.88–2.03) Other RR 1.85 (1.73–1.97)	RR 1.89 (1.71–2.09)		RR 3.75 (3.39–4.15)	RR 1.71 (1.43–2.03)	RR 1.98 (1.73–2.25)		RR 1.98 (1.73–2.27)	RR 1.33 (1.21–1.47)	RR 2.72 (2.00–3.68)		
Curkendall et al., 2006 [19]	Any RR 2.17 (2.00–2.33) Other OR 2.09 (1.99–2.20)	RR 1.49 (0.71–3.13) OR 1.83 (1.64–2.05)		RR 3.45 (2.78–4.17) OR 5.21 (4.86–5.58)	RR 1.67 (1.27–2.22) OR 2.09 (1.96–2.23)			RR 2.08 (1.52–2.86) OR 1.86 (1.72–2.01)	RR 1.23 (0.68–2.22) OR 1.24 (1.15–1.34)	RR 4.76 (0.79–25.00) OR 5.47 (4.25–7.03)		
Feary et al., 2010 [20]		≥75 y HR 1.34 (1.03–1.73) 35–44 y HR 10.34 (3.28–32.6)							≥75 y HR 1.10 (0.98–1.23) 35–44 y HR 3.44 (0.85–13.84)			
Schneider et al., 2010 [27]		OR 1.40 (1.13–1.73)			OR 1.19 (0.98–1.43)				OR 1.13 (0.92–1.38)	OR 2.51 (1.62–3.87)		
Rodriguez et al., 2010 [25]		RR 1.18 (0.81–1.71)		RR 2.81 (1.94–4.07) RR 2.94 (2.46–3.51)								
Sode et al., 2011 [28]		HR 1.26 (1.25–1.27)										
Baty et al., 2013 [29]		OR 1.80 (1.7–1.8)	OR 2.10 (2–2.2)	OR 3.20 (3.1–3.3) OR 2.70 (2.6–2.8)		OR 1.90 (1.9–2) OR 1.70 (1.7–1.8)					OR 5.80 (5.5–6.1) OR 5.60 (5–6.3)	OR 2.80 (2.7–2.9)
Cazzola et al., 2012 [21]		OR 2.91 (2.74–3.09)										
Garcia-Olmos et al., 2013 [30]			RR 1.41 (1.11–1.71)	RR 2.41 (1.88–2.93)	RR 1.25 (1.05–1.44)				RR 1.16 (0.89–1.42)			
Yin et al., 2014 [31]		HR 1.47 (1.41–1.55)										
Gershon et al., 2015 [32]	RR 1.94											
Ställberg et al., 2018 [35]		RR 2.25 (2.15–2.35)	RR 2.36 (2.28–2.45)	RR 3.27 (3.17–3.37)				RR 2.01 (1.94–2.09)	RR 1.71 (1.62–1.80) RRR 1.69 (1.53–1.87)	RR 2.39 (2.19–2.61)		
Morgan et al., 2018 [18]		>85 y HR 1.30 (1.12–1.52) 35–54 y HR 3.15 (2.64–3.76)		>85 y HR 1.51 (1.32–1.72) 35–54 y HR 6.80 (5.60–8.25)		>85 y HR 1.31 (1.17–1.47) 35–54 y HR 3.94 (3.22–4.81)	>85 y HR 1.87 (1.20–2.93) 35–54 y HR 4.06 (2.51–6.55)	>85 y HR 1.27 (1.06–1.53) 35–54 y HR 3.81 (3.35–4.34)	>85 y HR 0.96 (0.82–1.12) 35–54 y HR 3.81 (3.14–4.62)		>85 y HR 3.70 (2.20–6.23) 35–54 y HR 27.70 (15.33–50.06)	>85 y HR 1.42 (1.16–1.73) 35–54 y HR 7.70 (6.33–9.37)
Carter et al., 2019 [36]			OR 1.742 (1.688–1.797)	OR 2.167 (2.081–2.255)		OR 1.391 (1.339–1.444)			OR 0.973 (0.921–1.027)			OR 1.849 (1.737–1.969)
Ingebrigtsen et al., 2020 [37]			GOLD 1–2 HR 1.34 (1.20–1.49) GOLD 3–4 HR 1.80 (1.42–2.27)	GOLD 1–2 HR 1.91 (1.63–2.26) GOLD 3–4 HR 2.94 (2.22–3.88)								
Cave et al., 2021 [38]			RR 1.44 (1.35–1.55)	RR 2.64 (2.40–2.90)								
Jurevičienė et al., 2022 [39]	OR 1.83 (1.69–1.97)		OR 2.32 (2.14–2.50)	OR 2.61 (2.46–2.78)	OR 1.47 (1.38–1.55)							
Groenewegen et al., 2022 [40]			RR 1.69 (1.49–1.92)	RR 2.96 (2.58–3.40)		RR 1.56 (1.38–1.77)						
Maclagan et al., 2023 [23]		RR 1.88 (1.81–1.96)		RR 2.82 (2.70–2.91)		RR 1.79 (1.75–1.83)		RR 2.05 (1.94–2.18)	RR 1.71 (1.65–1.78)			

Abbreviations: AF, atrial fibrillation; CVD, cardiovascular disease; HF, heart failure; HR, hazard ratio; IHD, ischemic heart disease; MI, myocardial infarction; OR, odds ratio; RR, rate ratio/relative risk.

## Data Availability

All data analyzed during this review are derived from publicly available sources or published articles. No new data were created or analyzed in this study. The search strategy, including databases, search terms, and inclusion criteria, is detailed in the Materials and Methods section to ensure reproducibility and transparency in our review process.

## References

[1] Global Initiative for Chronic Obstructive Lung Disease (2024). Global Strategy for Prevention, Diagnosis and Management of Copd: 2024 Report. https://goldcopd.org/2024-gold-report/.

[2] Adeloye D., Song P., Zhu Y., Campbell H., Sheikh A., Rudan I. (2022). Global, regional, and national prevalence of, and risk factors for, chronic obstructive pulmonary disease (COPD) in 2019: A systematic review and modelling analysis. Lancet Respir. Med..

[3] Safiri S., Carson-Chahhoud K., Noori M., Nejadghaderi S.A., Sullman M.J.M., Heris J.A., Ansarin K., Mansournia M.A., Collins G.S., Kolahi A.-A. (2022). Burden of chronic obstructive pulmonary disease and its attributable risk factors in 204 countries and territories, 1990-2019: Results from the Global Burden of Disease Study 2019. BMJ.

[4] Kahnert K., Jörres R.A., Behr J., Welte T. (2023). The Diagnosis and Treatment of COPD and Its Comorbidities. Dtsch. Arztebl. Int..

[5] Polman R., Hurst J.R., Uysal O.F., Mandal S., Linz D., Simons S. (2024). Cardiovascular disease and risk in COPD: A state of the art review. Expert. Rev. Cardiovasc. Ther..

[6] Rogliani P., Ritondo B.L., Laitano R., Chetta A., Calzetta L. (2021). Advances in understanding of mechanisms related to increased cardiovascular risk in COPD. Expert. Rev. Respir. Med..

[7] Klaus F.R., John R.H., Samy S. (2018). Cardiovascular disease and COPD: Dangerous liaisons?. Eur. Respir. Rev..

[8] Divo M., Cote C., de Torres J.P., Casanova C., Marin J.M., Pinto-Plata V., Zulueta J., Cabrera C., Zagaceta J., Hunninghake G. (2012). Comorbidities and risk of mortality in patients with chronic obstructive pulmonary disease. Am. J. Respir. Crit. Care Med..

[9] Morgan A.D., Zakeri R., Quint J.K. (2018). Defining the relationship between COPD and CVD: What are the implications for clinical practice?. Ther. Adv. Respir. Dis..

[10] Chen W., Thomas J., Sadatsafavi M., FitzGerald J.M. (2015). Risk of cardiovascular comorbidity in patients with chronic obstructive pulmonary disease: A systematic review and meta-analysis. Lancet Respir. Med..

[11] Müllerova H., Agusti A., Erqou S., Mapel D.W. (2013). Cardiovascular comorbidity in COPD: Systematic literature review. Chest.

[12] Rothnie K.J., Yan R., Smeeth L., Quint J.K. (2015). Risk of myocardial infarction (MI) and death following MI in people with chronic obstructive pulmonary disease (COPD): A systematic review and meta-analysis. BMJ Open.

[13] Wang J.J. (2021). Risk of Coronary Heart Disease in People with Chronic Obstructive Pulmonary Disease: A Meta-Analysis. Int. J. Chron. Obs. Pulm. Dis..

[14] Ding C., Wang R., Gong X., Yuan Y. (2023). Stroke risk of COPD patients and death risk of COPD patients following a stroke: A systematic review and meta-analysis. Medicine.

[15] Müllerová H., Marshall J., de Nigris E., Varghese P., Pooley N., Embleton N., Nordon C., Marjenberg Z. (2022). Association of COPD exacerbations and acute cardiovascular events: A systematic review and meta-analysis. Ther. Adv. Respir. Dis..

[16] Page M.J., McKenzie J.E., Bossuyt P.M., Boutron I., Hoffmann T.C., Mulrow C.D., Shamseer L., Tetzlaff J.M., Akl E.A., Brennan S.E. (2021). The PRISMA 2020 statement: An updated guideline for reporting systematic reviews. PLoS Med..

[17] Huiart L., Ernst P., Suissa S. (2005). Cardiovascular morbidity and mortality in COPD. Chest.

[18] Morgan A.D., Rothnie K.J., Bhaskaran K., Smeeth L., Quint J.K. (2018). Chronic obstructive pulmonary disease and the risk of 12 cardiovascular diseases: A population-based study using UK primary care data. Thorax.

[19] Curkendall S.M., Deluise C., Jones J.K., Lanes S., Stang M.R., Goehring E., She D. (2006). Cardiovascular Disease in Patients with Chronic Obstructive Pulmonary Disease, Saskatchewan Canada: Cardiovascular Disease in COPD Patients. Ann. Epidemiol..

[20] Feary J.R., Rodrigues L.C., Smith C.J., Hubbard R.B., Gibson J.E. (2010). Prevalence of major comorbidities in subjects with COPD and incidence of myocardial infarction and stroke: A comprehensive analysis using data from primary care. Thorax.

[21] Cazzola M., Calzetta L., Bettoncelli G., Cricelli C., Romeo F., Matera M.G., Rogliani P. (2012). Cardiovascular disease in asthma and COPD: A population-based retrospective cross-sectional study. Respir. Med..

[22] Svendsen C.D., Kuiper K.K.J., Ostridge K., Larsen T.H., Nielsen R., Hodneland V., Nordeide E., Bakke P.S., Eagan T.M. (2022). Factors associated with coronary heart disease in COPD patients and controls. PLoS ONE.

[23] Maclagan L.C., Croxford R., Chu A., Sin D.D., Udell J.A., Lee D.S., Austin P.C., Gershon A.S. (2023). Quantifying COPD as a Risk Factor for Cardiac Disease in a Primary Prevention Cohort. Eur. Respir. J..

[24] Mattila T., Vasankari T., Kauppi P., Mazur W., Härkänen T., Heliövaara M. (2023). Mortality of asthma, COPD, and asthma-COPD overlap during an 18-year follow up. Respir. Med..

[25] Rodríguez L.A., Wallander M.A., Martín-Merino E., Johansson S. (2010). Heart failure, myocardial infarction, lung cancer and death in COPD patients: A UK primary care study. Respir. Med..

[26] Sidney S., Sorel M., Quesenberry C.P., DeLuise C., Lanes S., Eisner M.D. (2005). COPD and incident cardiovascular disease hospitalizations and mortality: Kaiser Permanente Medical Care Program. Chest.

[27] Schneider C., Bothner U., Jick S.S., Meier C.R. (2010). Chronic obstructive pulmonary disease and the risk of cardiovascular diseases. Eur. J. Epidemiol..

[28] Sode B.F., Dahl M., Nordestgaard B.G. (2011). Myocardial infarction and other co-morbidities in patients with chronic obstructive pulmonary disease: A Danish nationwide study of 7.4 million individuals. Eur. Heart J..

[29] Baty F., Putora P.M., Isenring B., Blum T., Brutsche M. (2013). Comorbidities and burden of COPD: A population based case-control study. PLoS ONE.

[30] García-Olmos L., Alberquilla Á., Ayala V., García-Sagredo P., Morales L., Carmona M., de Tena-Dávila M.J., Pascual M., Muñoz A., Salvador C.H. (2013). Comorbidity in patients with chronic obstructive pulmonary disease in family practice: A cross sectional study. BMC Fam. Pract..

[31] Yin L., Lensmar C., Ingelsson E., Bäck M. (2014). Differential association of chronic obstructive pulmonary disease with myocardial infarction and ischemic stroke in a nation-wide cohort. Int. J. Cardiol..

[32] Gershon A.S., Mecredy G.C., Guan J., Victor J.C., Goldstein R., To T. (2015). Quantifying comorbidity in individuals with COPD: A population study. Eur. Respir. J..

[33] Lahousse L., Niemeijer M.N., Berg M.E.v.D., Rijnbeek P.R., Joos G.F., Hofman A., Franco O.H., Deckers J.W., Eijgelsheim M., Stricker B.H. (2015). Chronic obstructive pulmonary disease and sudden cardiac death: The Rotterdam study. Eur. Heart J..

[34] Song S., Yang P.-S., Kim T.-H., Uhm J.-S., Pak H.-N., Lee M.-H., Joung B. (2017). Relation of Chronic Obstructive Pulmonary Disease to Cardiovascular Disease in the General Population. Am. J. Cardiol..

[35] Ställberg B., Janson C., Larsson K., Johansson G., Kostikas K., Gruenberger J.-B., Gutzwiller F.S., Jorgensen L., Uhde M., Lisspers K. (2018). Real-world retrospective cohort study ARCTIC shows burden of comorbidities in Swedish COPD versus non-COPD patients. NPJ Prim. Care Respir. Med..

[36] Carter P., Lagan J., Fortune C., Bhatt D.L., Vestbo J., Niven R., Chaudhuri N., Schelbert E.B., Potluri R., Miller C.A. (2019). Association of Cardiovascular Disease With Respiratory Disease. J. Am. Coll. Cardiol..

[37] Ingebrigtsen T.S., Marott J.L., Vestbo J., Nordestgaard B.G., Lange P. (2020). Coronary heart disease and heart failure in asthma, COPD and asthma-COPD overlap. BMJ Open Respir. Res..

[38] Cave A., Pham A., Lindeman C., Soos B., Williamson T., Drummond N. (2021). Chronic obstructive pulmonary disease as a risk factor in primary care: A Canadian retrospective cohort study. NPJ Prim. Care Respir. Med..

[39] Jurevičienė E., Burneikaitė G., Dambrauskas L., Kasiulevičius V., Kazėnaitė E., Navickas R., Puronaitė R., Smailytė G., Visockienė Ž., Danila E. (2022). Epidemiology of Chronic Obstructive Pulmonary Disease (COPD) Comorbidities in Lithuanian National Database: A Cluster Analysis. Int. J. Environ. Res. Public Health.

[40] Groenewegen A., Zwartkruis V.W., Smit L.J., A de Boer R., Rienstra M., Hoes A.W., Hollander M., Rutten F.H. (2022). Sex-specific and age-specific incidence of ischaemic heart disease, atrial fibrillation and heart failure in community patients with chronic obstructive pulmonary disease. BMJ Open Respir. Res..

[41] Zhao D. (2021). Epidemiological Features of Cardiovascular Disease in Asia. JACC Asia.

[42] Hurst J.R., Bhutani M., Bourbeau J., Han M., Hawkins N.M., Lam C.S., Marciniuk D.D., Price D., Stolz D., Zieroth S. (2024). MACE in COPD: Addressing cardiopulmonary risk. The Lancet Respir. Med..

[43] Shrikrishna D., Taylor C.J., Stonham C., Gale C.P. (2024). Exacerbating the burden of cardiovascular disease: How can we address cardiopulmonary risk in individuals with chronic obstructive pulmonary disease?. Eur. Heart J..

[44] Bafadhel M., Rabe K.F., Martinez F.J., Singh D., Darken P., Jenkins M., Aurivillius M., Patel M., Dorinsky P. (2022). Benefits of Budesonide/Glycopyrronium/Formoterol Fumarate Dihydrate on COPD Exacerbations, Lung Function, Symptoms, and Quality of Life Across Blood Eosinophil Ranges: A Post-Hoc Analysis of Data from ETHOS. Int. J. Chron. Obstruct Pulmon Dis..

[45] Tasha T., Desai A., Bajgain A., Ali A., Dutta C., Pasha K., Paul S., Abbas M.S., Nassar S.T., Mohammed L. (2023). A Literature Review on the Coexisting Chronic Obstructive Pulmonary Disease and Heart Failure. Cureus.

[46] Horodinschi R.N., Bratu O.G., Dediu G.N., Pantea Stoian A., Motofei I., Diaconu C.C. (2020). Heart failure and chronic obstructive pulmonary disease: A review. Acta Cardiol..

[47] Canepa M., Straburzynska-Migaj E., Drozdz J., Fernandez-Vivancos C., Pinilla J.M.G., Nyolczas N., Temporelli P.L., Mebazaa A., Lainscak M., Laroche C. (2018). Characteristics, treatments and 1-year prognosis of hospitalized and ambulatory heart failure patients with chronic obstructive pulmonary disease in the European Society of Cardiology Heart Failure Long-Term Registry. Eur. J. Heart Fail..

[48] Shahim B., Kapelios C.J., Savarese G., Lund L.H. (2023). Global Public Health Burden of Heart Failure: An Updated Review. Card. Fail. Rev..

[49] Quint J.K., Müllerova H., DiSantostefano R.L., Forbes H., Eaton S., Hurst J.R., Davis K., Smeeth L. (2014). Validation of chronic obstructive pulmonary disease recording in the Clinical Practice Research Datalink (CPRD-GOLD). BMJ Open.

[50] Williamson T., Green M.E., Birtwhistle R., Khan S., Garies S., Wong S.T., Natarajan N., Manca D., Drummond N. (2014). Validating the 8 CPCSSN case definitions for chronic disease surveillance in a primary care database of electronic health records. Ann. Fam. Med..

[51] Shanya S., Mohammad A.A., Ronan A.L., Jennifer K.Q., Gwyneth A.D. (2021). Identifying COPD in routinely collected electronic health records: A systematic scoping review. ERJ Open Res..

[52] Nathan S.D., Barbera J.A., Gaine S.P., Harari S., Martinez F.J., Olschewski H., Olsson K.M., Peacock A.J., Pepke-Zaba J., Provencher S. (2019). Pulmonary hypertension in chronic lung disease and hypoxia. Eur. Respir. J..

[53] Athlin Å., Lisspers K., Hasselgren M., Ställberg B., Janson C., Montgomery S., Giezeman M., Kisiel M., Nager A., Sandelowsky H. (2023). Diagnostic spirometry in COPD is increasing, a comparison of two Swedish cohorts. Npj Prim. Care Respir. Med..

[54] Gavina C., Carvalho D.S., Pardal M., Afonso-Silva M., Grangeia D., Dinis-Oliveira R.J., Araújo F., Taveira-Gomes T. (2022). Cardiovascular Risk Profile and Lipid Management in the Population-Based Cohort Study LATINO: 20 Years of Real-World Data. J. Clin. Med..

[55] Perone F., Bernardi M., Redheuil A., Mafrica D., Conte E., Spadafora L., Ecarnot F., Tokgozoglu L., Santos-Gallego C.G., Kaiser S.E. (2023). Role of Cardiovascular Imaging in Risk Assessment: Recent Advances, Gaps in Evidence, and Future Directions. J. Clin. Med..

[56] Swart K.M.A., Baak B.N., Lemmens L., Beest F.J.A.P.-V., Bengtsson C., Lobier M., Hoti F., Vojinovic D., van Burk L., Rhodes K. (2023). Risk of cardiovascular events after an exacerbation of chronic obstructive pulmonary disease: Results from the EXACOS-CV cohort study using the PHARMO Data Network in the Netherlands. Respir. Res..

